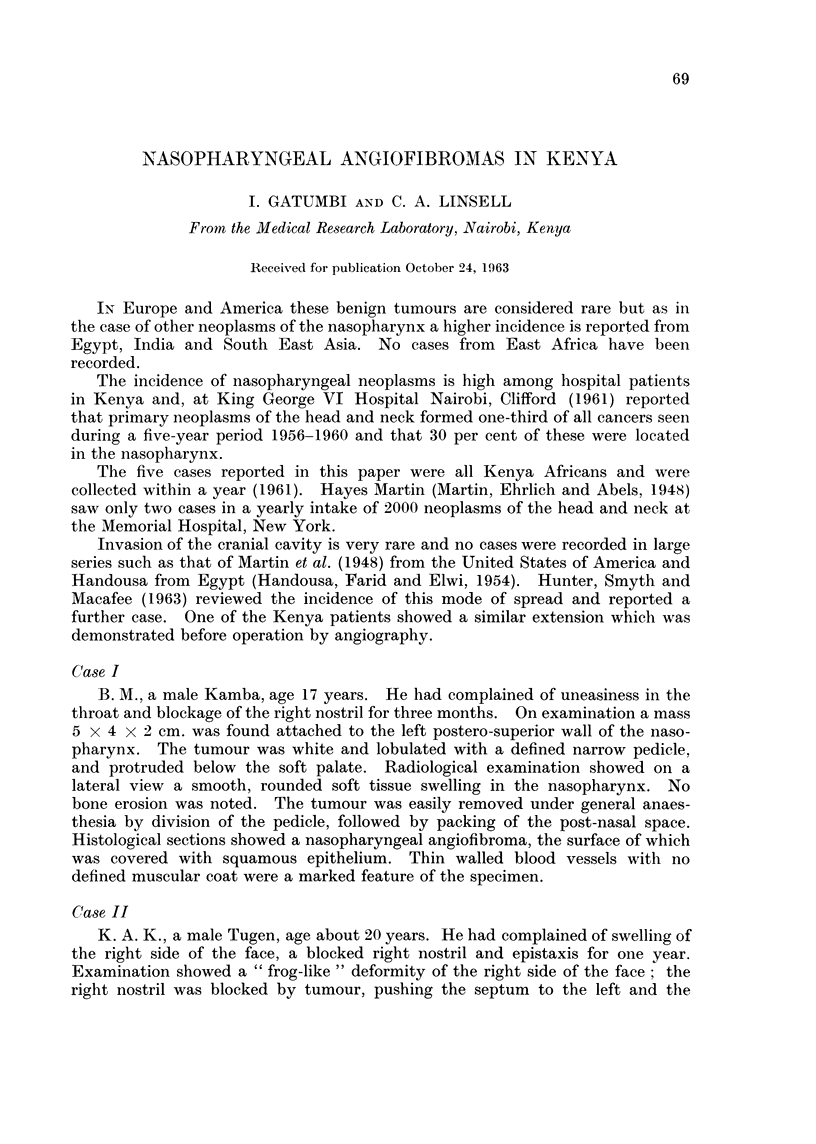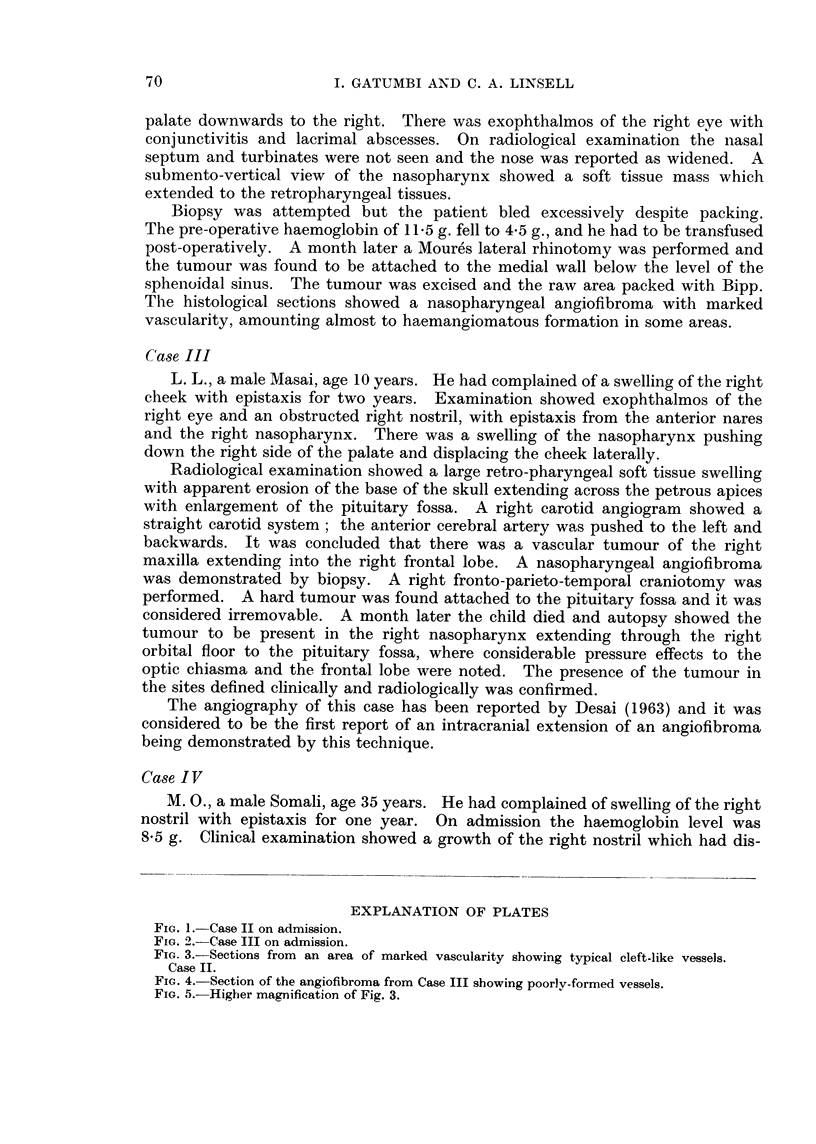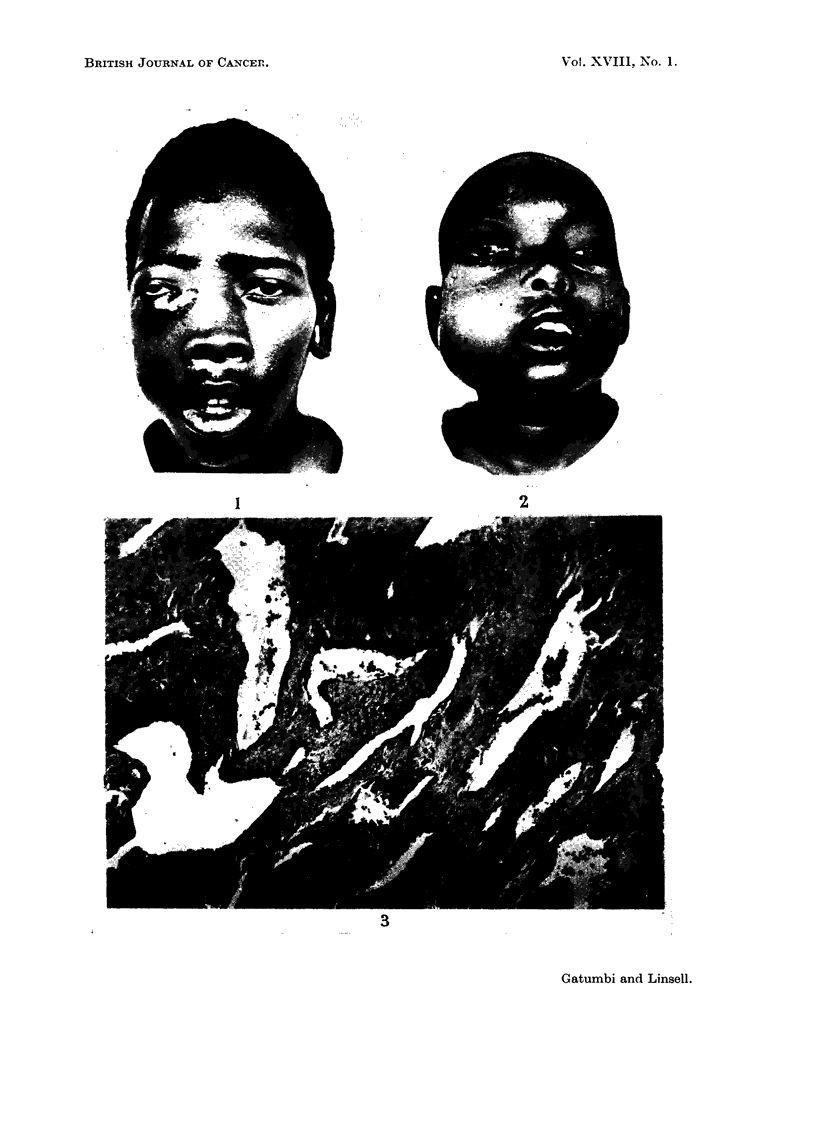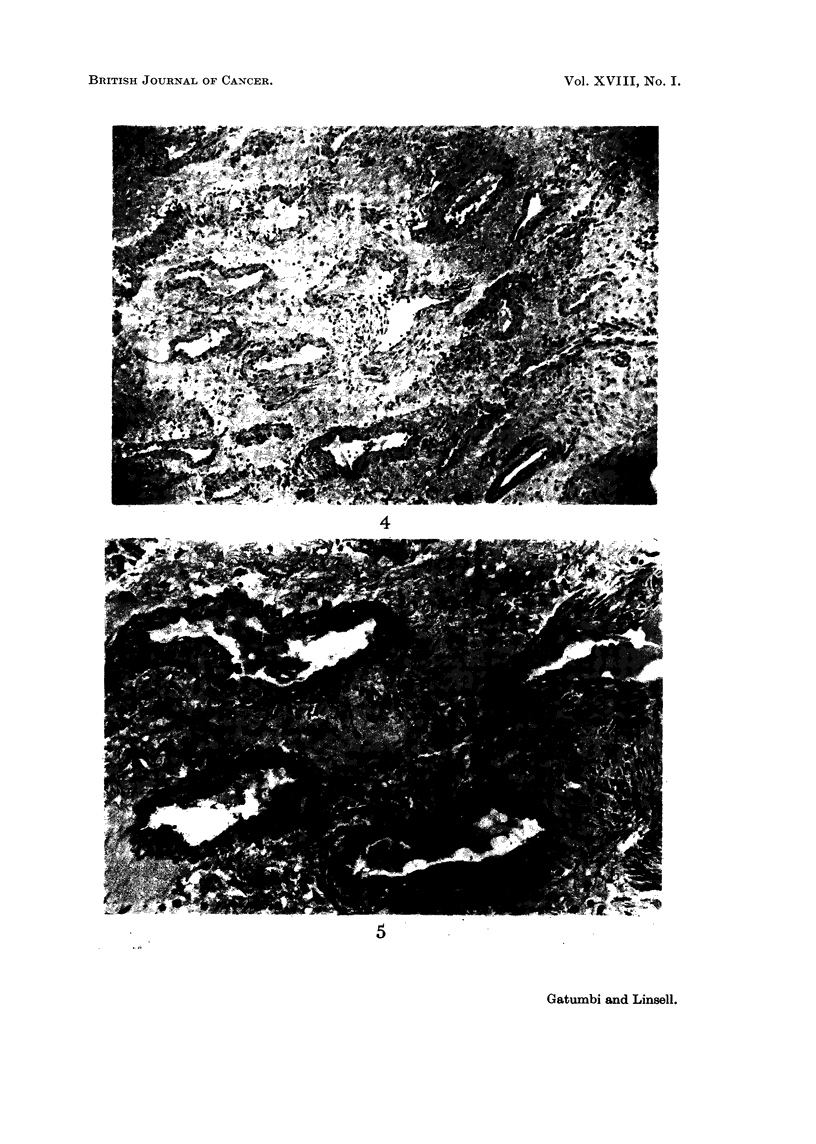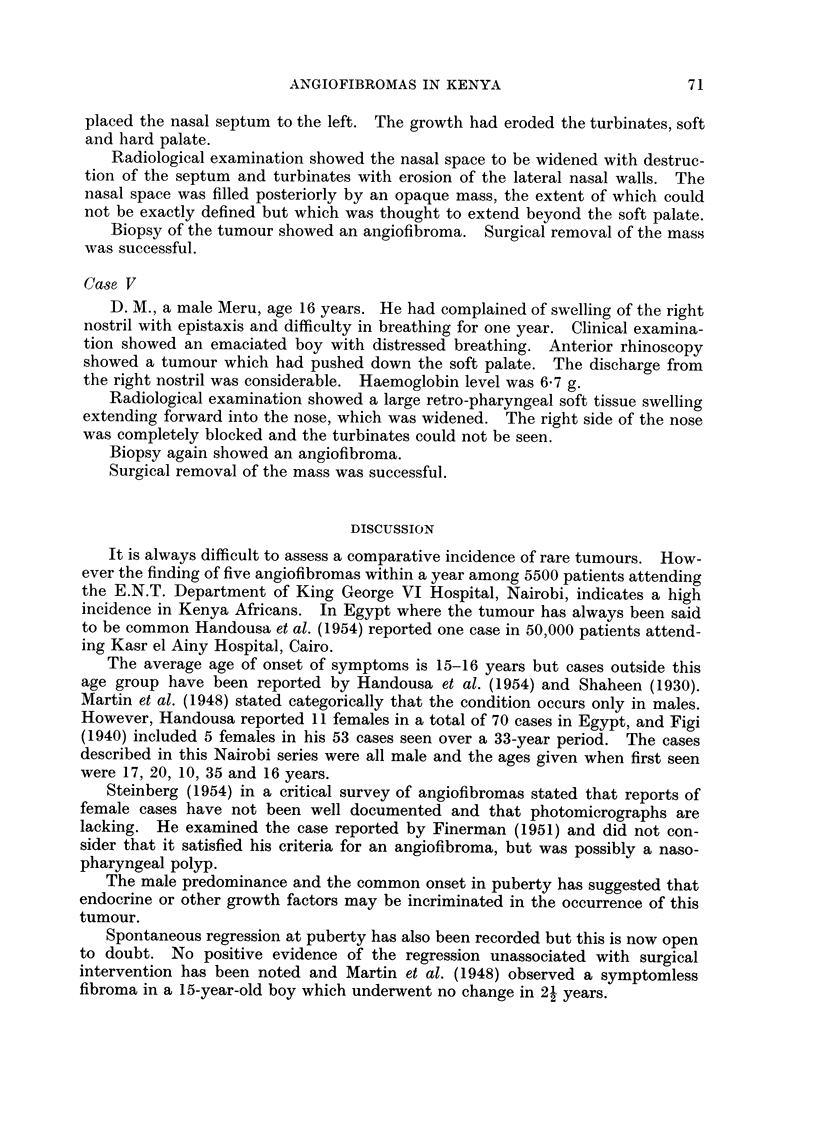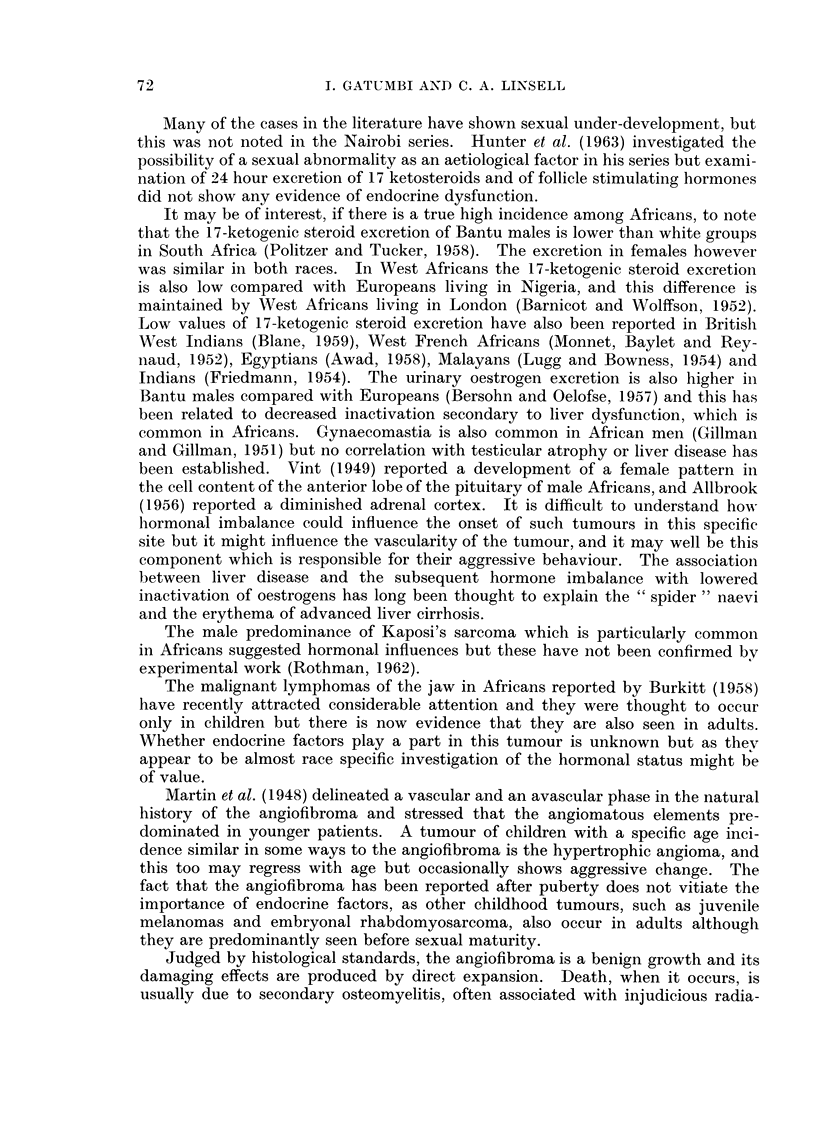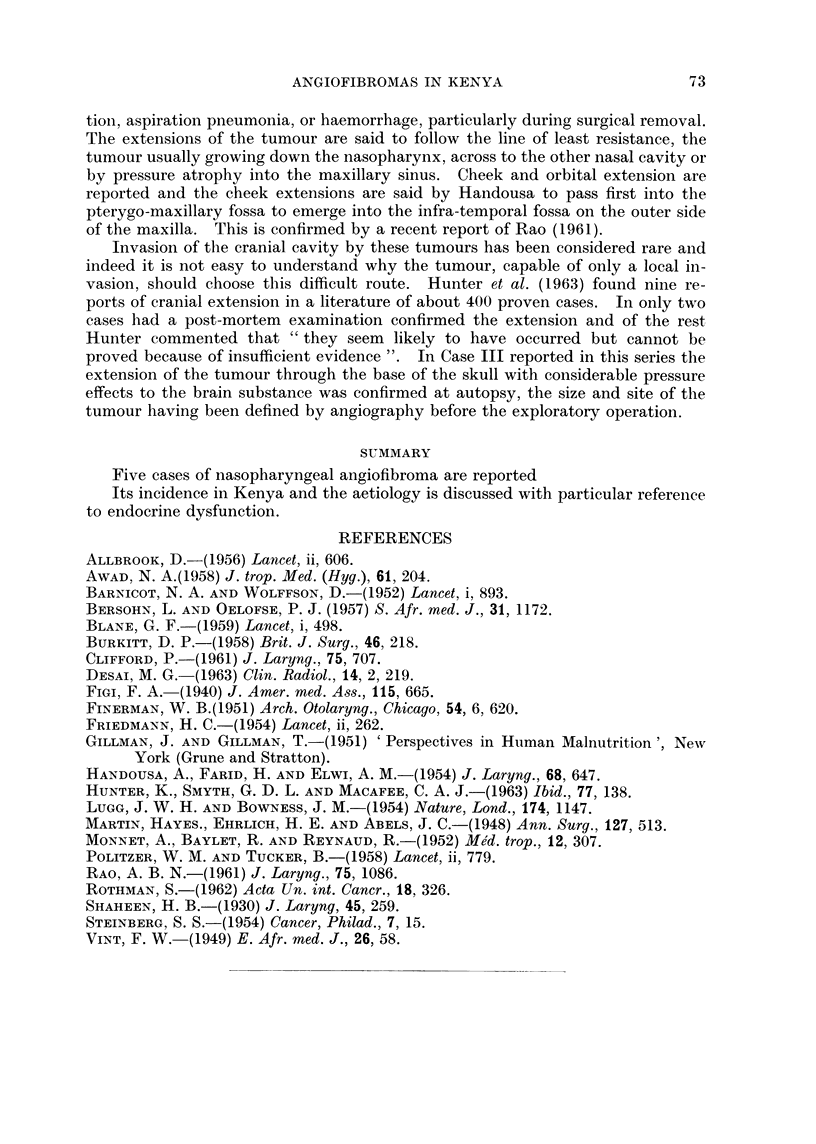# Nasopharyngeal Angiofibromas in Kenya

**DOI:** 10.1038/bjc.1964.7

**Published:** 1964-03

**Authors:** I. Gatumbi, C. A. Linsell

## Abstract

**Images:**


### NASOPHARYNGEAL ANGIOFIBROMAS IN KENYA

I. GATUMBI AND C. A. LINSELL

*From the Medical Research Laboratory, Nairobi, Kenya*

Received for publication October 24, 1963

IN Europe and America these benign tumours are considered rare but as in the case of other neoplasms of the nasopharynx a higher incidence is reported from Egypt, India and South East Asia. No cases from East Africa have been recorded.

The incidence of nasopharyngeal neoplasms is high among hospital patients in Kenya and, at King George VI Hospital Nairobi, Clifford (1961) reported that primary neoplasms of the head and neck formed one-third of all cancers seen during a five-year period 1956–1960 and that 30 per cent of these were located in the nasopharynx.

The five cases reported in this paper were all Kenya Africans and were collected within a year (1961). Hayes Martin (Martin, Ehrlich and Abels, 1948) saw only two cases in a yearly intake of 2000 neoplasms of the head and neck at the Memorial Hospital, New York.

Invasion of the cranial cavity is very rare and no cases were recorded in large series such as that of Martin *et al.* (1948) from the United States of America and Handousa from Egypt (Handousa, Farid and Elwi, 1954). Hunter, Smyth and Macafee (1963) reviewed the incidence of this mode of spread and reported a further case. One of the Kenya patients showed a similar extension which was demonstrated before operation by angiography.

*Case I*

B. M., a male Kamba, age 17 years. He had complained of uneasiness in the throat and blockage of the right nostril for three months. On examination a mass $5 \times 4 \times 2$ cm. was found attached to the left postero-superior wall of the nasopharynx. The tumour was white and lobulated with a defined narrow pedicle, and protruded below the soft palate. Radiological examination showed on a lateral view a smooth, rounded soft tissue swelling in the nasopharynx. No bone erosion was noted. The tumour was easily removed under general anaesthesia by division of the pedicle, followed by packing of the post-nasal space. Histological sections showed a nasopharyngeal angiofibroma, the surface of which was covered with squamous epithelium. Thin walled blood vessels with no defined muscular coat were a marked feature of the specimen.

*Case II*

K. A. K., a male Tugen, age about 20 years. He had complained of swelling of the right side of the face, a blocked right nostril and epistaxis for one year. Examination showed a " frog-like " deformity of the right side of the face ; the right nostril was blocked by tumour, pushing the septum to the left and the palate downwards to the right. There was exophthalmos of the right eye with conjunctivitis and lacrimal abscesses. On radiological examination the nasal septum and turbinates were not seen and the nose was reported as widened. A submento-vertical view of the nasopharynx showed a soft tissue mass which extended to the retropharyngeal tissues.

Biopsy was attempted but the patient bled excessively despite packing. The pre-operative haemoglobin of 11·5 g. fell to 4·5 g., and he had to be transfused post-operatively. A month later a Mourés lateral rhinotomy was performed and the tumour was found to be attached to the medial wall below the level of the sphenoidal sinus. The tumour was excised and the raw area packed with Bipp. The histological sections showed a nasopharyngeal angiofibroma with marked vascularity, amounting almost to haemangiomatous formation in some areas.

*Case III*

L. L., a male Masai, age 10 years. He had complained of a swelling of the right cheek with epistaxis for two years. Examination showed exophthalmos of the right eye and an obstructed right nostril, with epistaxis from the anterior nares and the right nasopharynx. There was a swelling of the nasopharynx pushing down the right side of the palate and displacing the cheek laterally.

Radiological examination showed a large retro-pharyngeal soft tissue swelling with apparent erosion of the base of the skull extending across the petrous apices with enlargement of the pituitary fossa. A right carotid angiogram showed a straight carotid system ; the anterior cerebral artery was pushed to the left and backwards. It was concluded that there was a vascular tumour of the right maxilla extending into the right frontal lobe. A nasopharyngeal angiofibroma was demonstrated by biopsy. A right fronto-parieto-temporal craniotomy was performed. A hard tumour was found attached to the pituitary fossa and it was considered irremovable. A month later the child died and autopsy showed the tumour to be present in the right nasopharynx extending through the right orbital floor to the pituitary fossa, where considerable pressure effects to the optic chiasma and the frontal lobe were noted. The presence of the tumour in the sites defined clinically and radiologically was confirmed.

The angiography of this case has been reported by Desai (1963) and it was considered to be the first report of an intracranial extension of an angiofibroma being demonstrated by this technique.

*Case IV*

M. O., a male Somali, age 35 years. He had complained of swelling of the right nostril with epistaxis for one year. On admission the haemoglobin level was 8·5 g. Clinical examination showed a growth of the right nostril which had dis-

---

EXPLANATION OF PLATES

FIG. 1.—Case II on admission.
FIG. 2.—Case III on admission.
FIG. 3.—Sections from an area of marked vascularity showing typical cleft-like vessels. Case II.
FIG. 4.—Section of the angiofibroma from Case III showing poorly-formed vessels.
FIG. 5.—Higher magnification of Fig. 3.

1

2

3

Gatumbi and Linsell.

4

5

Gatumbi and Linsell.

placed the nasal septum to the left. The growth had eroded the turbinates, soft and hard palate.

Radiological examination showed the nasal space to be widened with destruction of the septum and turbinates with erosion of the lateral nasal walls. The nasal space was filled posteriorly by an opaque mass, the extent of which could not be exactly defined but which was thought to extend beyond the soft palate.

Biopsy of the tumour showed an angiofibroma. Surgical removal of the mass was successful.

*Case V*

D. M., a male Meru, age 16 years. He had complained of swelling of the right nostril with epistaxis and difficulty in breathing for one year. Clinical examination showed an emaciated boy with distressed breathing. Anterior rhinoscopy showed a tumour which had pushed down the soft palate. The discharge from the right nostril was considerable. Haemoglobin level was 6·7 g.

Radiological examination showed a large retro-pharyngeal soft tissue swelling extending forward into the nose, which was widened. The right side of the nose was completely blocked and the turbinates could not be seen.

Biopsy again showed an angiofibroma.

Surgical removal of the mass was successful.

### DISCUSSION

It is always difficult to assess a comparative incidence of rare tumours. However the finding of five angiofibromas within a year among 5500 patients attending the E.N.T. Department of King George VI Hospital, Nairobi, indicates a high incidence in Kenya Africans. In Egypt where the tumour has always been said to be common Handousa *et al.* (1954) reported one case in 50,000 patients attending Kasr el Ainy Hospital, Cairo.

The average age of onset of symptoms is 15–16 years but cases outside this age group have been reported by Handousa *et al.* (1954) and Shaheen (1930). Martin *et al.* (1948) stated categorically that the condition occurs only in males. However, Handousa reported 11 females in a total of 70 cases in Egypt, and Figi (1940) included 5 females in his 53 cases seen over a 33-year period. The cases described in this Nairobi series were all male and the ages given when first seen were 17, 20, 10, 35 and 16 years.

Steinberg (1954) in a critical survey of angiofibromas stated that reports of female cases have not been well documented and that photomicrographs are lacking. He examined the case reported by Finerman (1951) and did not consider that it satisfied his criteria for an angiofibroma, but was possibly a nasopharyngeal polyp.

The male predominance and the common onset in puberty has suggested that endocrine or other growth factors may be incriminated in the occurrence of this tumour.

Spontaneous regression at puberty has also been recorded but this is now open to doubt. No positive evidence of the regression unassociated with surgical intervention has been noted and Martin *et al.* (1948) observed a symptomless fibroma in a 15-year-old boy which underwent no change in $2\frac{1}{2}$ years.

Many of the cases in the literature have shown sexual under-development, but this was not noted in the Nairobi series. Hunter *et al.* (1963) investigated the possibility of a sexual abnormality as an aetiological factor in his series but examination of 24 hour excretion of 17 ketosteroids and of follicle stimulating hormones did not show any evidence of endocrine dysfunction.

It may be of interest, if there is a true high incidence among Africans, to note that the 17-ketogenic steroid excretion of Bantu males is lower than white groups in South Africa (Politzer and Tucker, 1958). The excretion in females however was similar in both races. In West Africans the 17-ketogenic steroid excretion is also low compared with Europeans living in Nigeria, and this difference is maintained by West Africans living in London (Barnicot and Wolffson, 1952). Low values of 17-ketogenic steroid excretion have also been reported in British West Indians (Blane, 1959), West French Africans (Monnet, Baylet and Reynaud, 1952), Egyptians (Awad, 1958), Malayans (Lugg and Bowness, 1954) and Indians (Friedmann, 1954). The urinary oestrogen excretion is also higher in Bantu males compared with Europeans (Bersohn and Oelofse, 1957) and this has been related to decreased inactivation secondary to liver dysfunction, which is common in Africans. Gynaecomastia is also common in African men (Gillman and Gillman, 1951) but no correlation with testicular atrophy or liver disease has been established. Vint (1949) reported a development of a female pattern in the cell content of the anterior lobe of the pituitary of male Africans, and Allbrook (1956) reported a diminished adrenal cortex. It is difficult to understand how hormonal imbalance could influence the onset of such tumours in this specific site but it might influence the vascularity of the tumour, and it may well be this component which is responsible for their aggressive behaviour. The association between liver disease and the subsequent hormone imbalance with lowered inactivation of oestrogens has long been thought to explain the " spider " naevi and the erythema of advanced liver cirrhosis.

The male predominance of Kaposi's sarcoma which is particularly common in Africans suggested hormonal influences but these have not been confirmed by experimental work (Rothman, 1962).

The malignant lymphomas of the jaw in Africans reported by Burkitt (1958) have recently attracted considerable attention and they were thought to occur only in children but there is now evidence that they are also seen in adults. Whether endocrine factors play a part in this tumour is unknown but as they appear to be almost race specific investigation of the hormonal status might be of value.

Martin *et al.* (1948) delineated a vascular and an avascular phase in the natural history of the angiofibroma and stressed that the angiomatous elements predominated in younger patients. A tumour of children with a specific age incidence similar in some ways to the angiofibroma is the hypertrophic angioma, and this too may regress with age but occasionally shows aggressive change. The fact that the angiofibroma has been reported after puberty does not vitiate the importance of endocrine factors, as other childhood tumours, such as juvenile melanomas and embryonal rhabdomyosarcoma, also occur in adults although they are predominantly seen before sexual maturity.

Judged by histological standards, the angiofibroma is a benign growth and its damaging effects are produced by direct expansion. Death, when it occurs, is usually due to secondary osteomyelitis, often associated with injudicious radiation, aspiration pneumonia, or haemorrhage, particularly during surgical removal. The extensions of the tumour are said to follow the line of least resistance, the tumour usually growing down the nasopharynx, across to the other nasal cavity or by pressure atrophy into the maxillary sinus. Cheek and orbital extension are reported and the cheek extensions are said by Handousa to pass first into the pterygo-maxillary fossa to emerge into the infra-temporal fossa on the outer side of the maxilla. This is confirmed by a recent report of Rao (1961).

Invasion of the cranial cavity by these tumours has been considered rare and indeed it is not easy to understand why the tumour, capable of only a local invasion, should choose this difficult route. Hunter *et al.* (1963) found nine reports of cranial extension in a literature of about 400 proven cases. In only two cases had a post-mortem examination confirmed the extension and of the rest Hunter commented that " they seem likely to have occurred but cannot be proved because of insufficient evidence ". In Case III reported in this series the extension of the tumour through the base of the skull with considerable pressure effects to the brain substance was confirmed at autopsy, the size and site of the tumour having been defined by angiography before the exploratory operation.

### SUMMARY

Five cases of nasopharyngeal angiofibroma are reported

Its incidence in Kenya and the aetiology is discussed with particular reference to endocrine dysfunction.

### REFERENCES

Allbrook, D.—(1956) *Lancet*, ii, 606.
Awad, N. A.(1958) *J. trop. Med.* (*Hyg.*), **61**, 204.
Barnicot, N. A. and Wolffson, D.—(1952) *Lancet*, i, 893.
Bersohn, L. and Oelofse, P. J. (1957) *S. Afr. med. J.*, **31**, 1172.
Blane, G. F.—(1959) *Lancet*, i, 498.
Burkitt, D. P.—(1958) *Brit. J. Surg.*, **46**, 218.
Clifford, P.—(1961) *J. Laryng.*, **75**, 707.
Desai, M. G.—(1963) *Clin. Radiol.*, **14**, 2, 219.
Figi, F. A.—(1940) *J. Amer. med. Ass.*, **115**, 665.
Finerman, W. B.(1951) *Arch. Otolaryng., Chicago*, **54**, 6, 620.
Friedmann, H. C.—(1954) *Lancet*, ii, 262.
Gillman, J. and Gillman, T.—(1951) ' Perspectives in Human Malnutrition ', New York (Grune and Stratton).
Handousa, A., Farid, H. and Elwi, A. M.—(1954) *J. Laryng.*, **68**, 647.
Hunter, K., Smyth, G. D. L. and Macafee, C. A. J.—(1963) *Ibid.*, **77**, 138.
Lugg, J. W. H. and Bowness, J. M.—(1954) *Nature, Lond.*, **174**, 1147.
Martin, Hayes., Ehrlich, H. E. and Abels, J. C.—(1948) *Ann. Surg.*, **127**, 513.
Monnet, A., Baylet, R. and Reynaud, R.—(1952) *Méd. trop.*, **12**, 307.
Politzer, W. M. and Tucker, B.—(1958) *Lancet*, ii, 779.
Rao, A. B. N.—(1961) *J. Laryng.*, **75**, 1086.
Rothman, S.—(1962) *Acta Un. int. Cancr.*, **18**, 326.
Shaheen, H. B.—(1930) *J. Laryng*, **45**, 259.
Steinberg, S. S.—(1954) *Cancer, Philad.*, **7**, 15.
Vint, F. W.—(1949) *E. Afr. med. J.*, **26**, 58.